# Bis(2-ethyl-1*H*-imidazol-3-ium) tetra­chloridocuprate(II)

**DOI:** 10.1107/S160053681005261X

**Published:** 2010-12-24

**Authors:** Run-Qiang Zhu

**Affiliations:** a Ordered Matter Science Research Center, College of Chemistry and Chemical Engineering, Southeast University, Nanjing 211189, People’s Republic of China

## Abstract

In the crystal structure of the title salt, (C_5_H_9_N_2_)_2_[CuCl_4_], the organic cations and the tetrahedral [CuCl_4_] anions are linked into a three-dimensional network by N—H⋯Cl hydrogen bonds. The two 2-ethyl imidazolium cations in the asymmetric unit differ in the orientation of the ethyl group, with N—C—C—C torsion angles of −170.0 (4) and −87.6 (5)°.

## Related literature

For general background to ferroelectric metal-organic frameworks, see: Fu *et al.* (2009 ▶); Ye *et al.* (2006 ▶); Zhang *et al.* (2008 ▶, 2010 ▶).
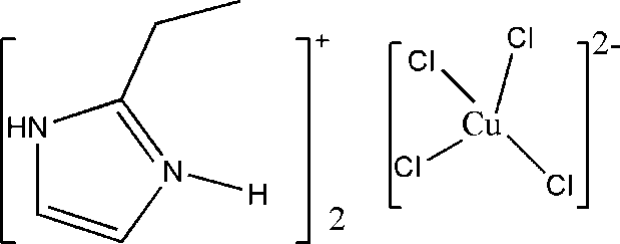


## Experimental

### 

#### Crystal data

(C_5_H_9_N_2_)_2_[CuCl_4_]
*M* *_r_* = 399.63
Triclinic, 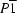

*a* = 7.992 (4) Å
*b* = 9.003 (4) Å
*c* = 12.216 (6) Å
α = 79.641 (14)°
β = 84.646 (14)°
γ = 72.154 (12)°
*V* = 822.4 (7) Å^3^
*Z* = 2
Mo *K*α radiation
μ = 1.97 mm^−1^
*T* = 293 K
0.30 × 0.25 × 0.20 mm

#### Data collection

Rigaku SCXmini diffractometer
Absorption correction: multi-scan (*CrystalClear*; Rigaku, 2005 ▶) *T*_min_ = 0.559, *T*_max_ = 0.674
9065 measured reflections
3775 independent reflections
3124 reflections with *I* > 2σ(*I*)
*R*_int_ = 0.035

#### Refinement

*R*[*F* ^2^ > 2σ(*F* ^2^)] = 0.039
*wR*(*F* ^2^) = 0.123
*S* = 1.17
3775 reflections
174 parameters
H-atom parameters constrained
Δρ_max_ = 0.59 e Å^−3^
Δρ_min_ = −0.66 e Å^−3^

### 

Data collection: *CrystalClear* (Rigaku, 2005 ▶); cell refinement: *CrystalClear*; data reduction: *CrystalClear*; program(s) used to solve structure: *SHELXS97* (Sheldrick, 2008 ▶); program(s) used to refine structure: *SHELXL97* (Sheldrick, 2008 ▶); molecular graphics: *SHELXTL* (Sheldrick, 2008 ▶); software used to prepare material for publication: *SHELXL97*.

## Supplementary Material

Crystal structure: contains datablocks I, global. DOI: 10.1107/S160053681005261X/vm2067sup1.cif

Structure factors: contains datablocks I. DOI: 10.1107/S160053681005261X/vm2067Isup2.hkl

Additional supplementary materials:  crystallographic information; 3D view; checkCIF report

## Figures and Tables

**Table 1:** Hydrogen-bond geometry (Å, °)

*D*—H⋯*A*	*D*—H	H⋯*A*	*D*⋯*A*	*D*—H⋯*A*
N1—H1*A*⋯Cl1^i^	0.86	2.39	3.217 (3)	160
N2—H2*A*⋯Cl2	0.86	2.39	3.195 (3)	157
N3—H3*A*⋯Cl3	0.86	2.46	3.178 (3)	142
N4—H4*C*⋯Cl4^ii^	0.86	2.32	3.149 (3)	164

Symmetry codes: (i) 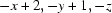; (ii) 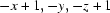.

**Fig. 1.**
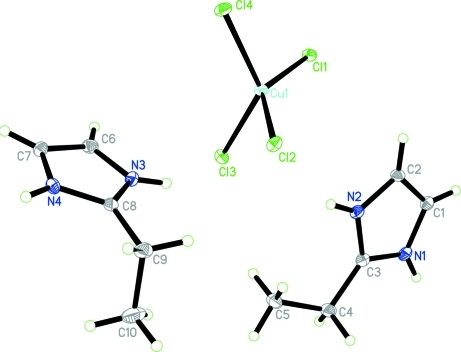
The molecular structure of the title compound, with the displacement ellipsoids drawn at the 30% probability level. Intramolecular hydrogen bonds are shown as dashed lines.

**Fig. 2.**
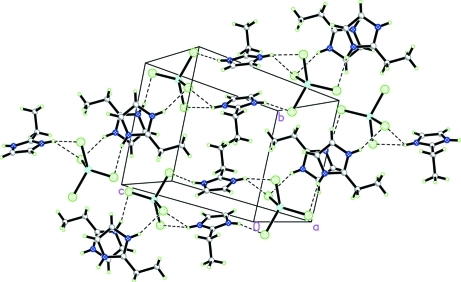
Packing diagram of the title compound, showing the structure along the b axis. Hydrogen bonds are shown as dashed lines.

## supplementary crystallographic information

### Comment

Dielectric constant measurements of compounds as a function of temperature is the basic method to find the materials which possess potential ferroelectric phase changes (Fu *et al.*, 2009; Ye *et al.*, 2006; Zhang *et al.*, 2008; Zhang *et al.*, 2010). The dielectric constant of the title compound has been measured, but showed no dielectric disuniformity in the range 93–365 K (m.p. 374–381 K).

X-ray crystallographic studies have been carried out for the complex 2[C_5_N_2_H_9_] ^+^.CuCl_4_^2-^ at 123 K. An view of the complex is shown in Fig. 1. The structure is consolidated by extensive intermolecular and intramolecular hydrogen bonds between Cl and N. This hydrogen bonding (Table 1, Fig. 2) produces a three-dimensional network. Within the CuCl_4_^2-^ tetrahedra the Cu-Cl distances are: Cu1—Cl1 = 2.2287 (13) Å, Cu1—Cl2 = 2.2625 Å,Cu1—Cl3 = 2.2688 (12) Å, Cu1—Cl4 = 2.2501 (13) Å.

The two 2-ethyl imidazolium cations in the asymmetric unit differ in the orientation of the ethyl group. In one cation all atoms except H atoms are situated in the same plane(dihedral angle N1—C3—C4—C5 = -170.0 (4)°), while in the other cation the dihedral angle N3—C8—C9—C10 is -87.6 (5) °.

### Experimental

A mixture of CuCl_2_ (4.26 g, 25 mmol), hydrochloric acid (50 mmol), and 2-ethyl imidazole (4.8 g, 50 mmol) in water was stirred for several days at room temperature, yellow block crystals were obtained.

### Refinement

Hydrogen atom positions were calculated and allowed to ride on their respective C atoms and N atoms with C–H distances of 0.93–0.97Å and N–H = 0.86 Å, and with U_iso_(H)=1.2U_eq_(C or N).

### Crystal data

**Table:**

(C_5_H_9_N_2_)_2_[CuCl_4_]	*Z* = 2
*M**_r_* = 399.63	*F*(000) = 406
Triclinic, *P*1	*D*_x_ = 1.614 Mg m^−^^3^
Hall symbol: -P 1	Mo *K*α radiation, λ = 0.71073 Å
*a* = 7.992 (4) Å	Cell parameters from 2088 reflections
*b* = 9.003 (4) Å	θ = 2.4–27.5°
*c* = 12.216 (6) Å	µ = 1.97 mm^−^^1^
α = 79.641 (14)°	*T* = 293 K
β = 84.646 (14)°	Block, yellow
γ = 72.154 (12)°	0.30 × 0.25 × 0.20 mm
*V* = 822.4 (7) Å^3^	

### Data collection

**Table:**

Rigaku SCXmini diffractometer	3775 independent reflections
Radiation source: fine-focus sealed tube	3124 reflections with *I* > 2σ(*I*)
graphite	*R*_int_ = 0.035
CCD_Profile_fitting scans	θ_max_ = 27.5°, θ_min_ = 1.7°
Absorption correction: multi-scan (*CrystalClear*; Rigaku, 2005)	*h* = −10→10
*T*_min_ = 0.559, *T*_max_ = 0.674	*k* = −11→11
9065 measured reflections	*l* = −15→15

### Refinement

**Table:**

Refinement on *F*^2^	Secondary atom site location: difference Fourier map
Least-squares matrix: full	Hydrogen site location: inferred from neighbouring sites
*R*[*F*^2^ > 2σ(*F*^2^)] = 0.039	H-atom parameters constrained
*wR*(*F*^2^) = 0.123	*w* = 1/[σ^2^(*F*_o_^2^) + (0.0636*P*)^2^] where *P* = (*F*_o_^2^ + 2*F*_c_^2^)/3
*S* = 1.17	(Δ/σ)_max_ = 0.001
3775 reflections	Δρ_max_ = 0.59 e Å^−^^3^
174 parameters	Δρ_min_ = −0.66 e Å^−^^3^
0 restraints	Extinction correction: *SHELXL97* (Sheldrick, 2008)
Primary atom site location: structure-invariant direct methods	Extinction coefficient: 0.0014 (1)

### Special details

**Table:**

Geometry. All e.s.d.'s (except the e.s.d. in the dihedral angle between two l.s. planes) are estimated using the full covariance matrix. The cell e.s.d.'s are taken into account individually in the estimation of e.s.d.'s in distances, angles and torsion angles; correlations between e.s.d.'s in cell parameters are only used when they are defined by crystal symmetry. An approximate (isotropic) treatment of cell e.s.d.'s is used for estimating e.s.d.'s involving l.s. planes.
Refinement. Refinement of *F*^2^ against ALL reflections. The weighted *R*-factor *wR* and goodness of fit *S* are based on *F*^2^, conventional *R*-factors *R* are based on *F*, with *F* set to zero for negative *F*^2^. The threshold expression of *F*^2^ > σ(*F*^2^) is used only for calculating *R*-factors(gt) *etc*. and is not relevant to the choice of reflections for refinement. *R*-factors based on *F*^2^ are statistically about twice as large as those based on *F*, and *R*- factors based on ALL data will be even larger.

### Fractional atomic coordinates and isotropic or equivalent isotropic displacement parameters (Å^2^)

**Table:**

	*x*	*y*	*z*	*U*_iso_*/*U*_eq_	
Cu1	0.91120 (5)	0.05748 (5)	0.21071 (3)	0.01639 (14)	
C1	0.8790 (5)	0.4965 (4)	−0.1245 (3)	0.0203 (7)	
H1	0.9490	0.4905	−0.1897	0.024*	
C2	0.8332 (5)	0.3759 (4)	−0.0584 (3)	0.0192 (7)	
H2	0.8657	0.2709	−0.0691	0.023*	
C3	0.7099 (4)	0.5947 (4)	0.0167 (3)	0.0183 (7)	
C4	0.6042 (5)	0.7072 (4)	0.0911 (3)	0.0250 (8)	
H4A	0.5023	0.7782	0.0523	0.030*	
H4B	0.6747	0.7704	0.1076	0.030*	
C5	0.5432 (5)	0.6239 (5)	0.1994 (3)	0.0285 (9)	
H5A	0.4760	0.5588	0.1835	0.043*	
H5B	0.4713	0.7010	0.2426	0.043*	
H5C	0.6436	0.5591	0.2406	0.043*	
C6	0.7322 (5)	0.0989 (4)	0.5488 (3)	0.0223 (8)	
H6	0.8545	0.0669	0.5483	0.027*	
C7	0.6228 (5)	0.0601 (4)	0.6319 (3)	0.0208 (7)	
H7	0.6544	−0.0045	0.6998	0.025*	
C8	0.4588 (5)	0.2168 (4)	0.4944 (3)	0.0177 (7)	
C9	0.3058 (5)	0.3210 (4)	0.4307 (3)	0.0257 (8)	
H9A	0.2071	0.2783	0.4487	0.031*	
H9B	0.3347	0.3231	0.3517	0.031*	
C10	0.2529 (6)	0.4888 (5)	0.4567 (4)	0.0397 (11)	
H10A	0.2191	0.4877	0.5343	0.060*	
H10B	0.1557	0.5535	0.4126	0.060*	
H10C	0.3507	0.5311	0.4397	0.060*	
Cl1	1.16150 (11)	0.01933 (9)	0.10773 (7)	0.01903 (19)	
Cl2	0.61789 (11)	0.16204 (10)	0.18983 (7)	0.0194 (2)	
Cl3	0.94259 (11)	0.24838 (10)	0.29860 (7)	0.0204 (2)	
Cl4	0.91629 (11)	−0.19331 (10)	0.27848 (8)	0.0233 (2)	
N1	0.8018 (4)	0.6294 (3)	−0.0763 (2)	0.0193 (6)	
H1A	0.8113	0.7219	−0.1024	0.023*	
N2	0.7279 (4)	0.4409 (3)	0.0285 (2)	0.0187 (6)	
H2A	0.6811	0.3891	0.0823	0.022*	
N3	0.6284 (4)	0.1948 (3)	0.4648 (2)	0.0201 (6)	
H3A	0.6673	0.2350	0.4020	0.024*	
N4	0.4552 (4)	0.1350 (3)	0.5963 (2)	0.0186 (6)	
H4C	0.3608	0.1298	0.6347	0.022*	

### Atomic displacement parameters (Å^2^)

**Table:**

	*U*^11^	*U*^22^	*U*^33^	*U*^12^	*U*^13^	*U*^23^
Cu1	0.0145 (2)	0.0147 (2)	0.0195 (2)	−0.00468 (17)	0.00088 (16)	−0.00158 (16)
C1	0.0197 (18)	0.0200 (17)	0.0211 (18)	−0.0069 (14)	−0.0028 (14)	−0.0008 (14)
C2	0.0228 (18)	0.0141 (16)	0.0215 (18)	−0.0061 (14)	0.0004 (14)	−0.0046 (13)
C3	0.0176 (17)	0.0170 (16)	0.0219 (18)	−0.0063 (14)	−0.0059 (14)	−0.0029 (13)
C4	0.029 (2)	0.0196 (18)	0.027 (2)	−0.0036 (16)	−0.0039 (16)	−0.0077 (15)
C5	0.029 (2)	0.029 (2)	0.025 (2)	0.0003 (17)	−0.0018 (16)	−0.0111 (16)
C6	0.0176 (17)	0.0208 (18)	0.026 (2)	−0.0031 (14)	0.0002 (15)	−0.0040 (15)
C7	0.0209 (18)	0.0199 (17)	0.0191 (18)	−0.0021 (14)	−0.0051 (14)	−0.0012 (14)
C8	0.0213 (18)	0.0142 (16)	0.0181 (17)	−0.0053 (14)	0.0002 (14)	−0.0046 (13)
C9	0.0232 (19)	0.0235 (19)	0.028 (2)	0.0009 (16)	−0.0101 (16)	−0.0062 (16)
C10	0.042 (3)	0.023 (2)	0.048 (3)	0.0048 (19)	−0.020 (2)	−0.0072 (19)
Cl1	0.0178 (4)	0.0166 (4)	0.0227 (4)	−0.0061 (3)	0.0043 (3)	−0.0042 (3)
Cl2	0.0147 (4)	0.0207 (4)	0.0209 (4)	−0.0052 (3)	−0.0015 (3)	0.0019 (3)
Cl3	0.0213 (4)	0.0221 (4)	0.0206 (4)	−0.0103 (3)	0.0030 (3)	−0.0061 (3)
Cl4	0.0208 (4)	0.0152 (4)	0.0293 (5)	−0.0035 (3)	0.0054 (4)	0.0017 (3)
N1	0.0217 (15)	0.0157 (14)	0.0206 (15)	−0.0080 (12)	−0.0038 (12)	0.0027 (12)
N2	0.0195 (15)	0.0158 (14)	0.0195 (15)	−0.0052 (12)	−0.0007 (12)	0.0006 (11)
N3	0.0221 (16)	0.0188 (14)	0.0177 (15)	−0.0058 (12)	0.0012 (12)	0.0000 (12)
N4	0.0161 (14)	0.0221 (15)	0.0181 (15)	−0.0067 (12)	0.0034 (12)	−0.0048 (12)

### Geometric parameters (Å, °)

**Table:**

Cu1—Cl1	2.2287 (13)	C6—C7	1.346 (5)
Cu1—Cl4	2.2501 (13)	C6—N3	1.374 (4)
Cu1—Cl2	2.2625 (14)	C6—H6	0.9300
Cu1—Cl3	2.2688 (12)	C7—N4	1.374 (4)
C1—C2	1.355 (5)	C7—H7	0.9300
C1—N1	1.375 (5)	C8—N4	1.330 (4)
C1—H1	0.9300	C8—N3	1.333 (4)
C2—N2	1.391 (4)	C8—C9	1.481 (5)
C2—H2	0.9300	C9—C10	1.523 (5)
C3—N2	1.330 (4)	C9—H9A	0.9700
C3—N1	1.335 (5)	C9—H9B	0.9700
C3—C4	1.493 (5)	C10—H10A	0.9600
C4—C5	1.516 (6)	C10—H10B	0.9600
C4—H4A	0.9700	C10—H10C	0.9600
C4—H4B	0.9700	N1—H1A	0.8600
C5—H5A	0.9600	N2—H2A	0.8600
C5—H5B	0.9600	N3—H3A	0.8600
C5—H5C	0.9600	N4—H4C	0.8600
			
Cl1—Cu1—Cl4	101.08 (4)	C6—C7—N4	106.2 (3)
Cl1—Cu1—Cl2	139.56 (4)	C6—C7—H7	126.9
Cl4—Cu1—Cl2	98.32 (4)	N4—C7—H7	126.9
Cl1—Cu1—Cl3	97.91 (4)	N4—C8—N3	105.9 (3)
Cl4—Cu1—Cl3	130.13 (5)	N4—C8—C9	126.9 (3)
Cl2—Cu1—Cl3	96.09 (4)	N3—C8—C9	127.0 (3)
C2—C1—N1	106.7 (3)	C8—C9—C10	111.8 (3)
C2—C1—H1	126.7	C8—C9—H9A	109.3
N1—C1—H1	126.7	C10—C9—H9A	109.3
C1—C2—N2	106.1 (3)	C8—C9—H9B	109.3
C1—C2—H2	127.0	C10—C9—H9B	109.3
N2—C2—H2	127.0	H9A—C9—H9B	107.9
N2—C3—N1	106.5 (3)	C9—C10—H10A	109.5
N2—C3—C4	126.7 (3)	C9—C10—H10B	109.5
N1—C3—C4	126.8 (3)	H10A—C10—H10B	109.5
C3—C4—C5	112.6 (3)	C9—C10—H10C	109.5
C3—C4—H4A	109.1	H10A—C10—H10C	109.5
C5—C4—H4A	109.1	H10B—C10—H10C	109.5
C3—C4—H4B	109.1	C3—N1—C1	110.5 (3)
C5—C4—H4B	109.1	C3—N1—H1A	124.7
H4A—C4—H4B	107.8	C1—N1—H1A	124.7
C4—C5—H5A	109.5	C3—N2—C2	110.3 (3)
C4—C5—H5B	109.5	C3—N2—H2A	124.9
H5A—C5—H5B	109.5	C2—N2—H2A	124.9
C4—C5—H5C	109.5	C8—N3—C6	110.3 (3)
H5A—C5—H5C	109.5	C8—N3—H3A	124.8
H5B—C5—H5C	109.5	C6—N3—H3A	124.8
C7—C6—N3	106.8 (3)	C8—N4—C7	110.8 (3)
C7—C6—H6	126.6	C8—N4—H4C	124.6
N3—C6—H6	126.6	C7—N4—H4C	124.6

### Hydrogen-bond geometry (Å, °)

**Table:**

*D*—H···*A*	*D*—H	H···*A*	*D*···*A*	*D*—H···*A*
N1—H1A···Cl1^i^	0.86	2.39	3.217 (3)	160
N2—H2A···Cl2	0.86	2.39	3.195 (3)	157
N3—H3A···Cl3	0.86	2.46	3.178 (3)	142
N4—H4C···Cl4^ii^	0.86	2.32	3.149 (3)	164

Symmetry codes: (i) −*x*+2, −*y*+1, −*z*; (ii) −*x*+1, −*y*, −*z*+1.

## References

[bb1] Fu, D.-W., Ge, J.-Z., Dai, J., Ye, H.-Y. & Qu, Z.-R. (2009). *Inorg. Chem. Commun.* **12**, 994–997.

[bb2] Rigaku (2005). *CrystalClear* Rigaku Corporation, Tokyo, Japan.

[bb3] Sheldrick, G. M. (2008). *Acta Cryst.* A**64**, 112–122. 10.1107/S0108767307043930 18156677

[bb4] Ye, Q., Song, Y.-M., Wang, G.-X., Chen, K. & Fu, D.-W. (2006). *J. Am. Chem. Soc.* **128**, 6554–6555. 10.1021/ja060856p 16704244

[bb5] Zhang, W., Xiong, R.-G. & Huang, S.-P. D. (2008). *J. Am. Chem. Soc.* **130**, 10468–10469. 10.1021/ja803021v 18636707

[bb6] Zhang, W., Ye, H.-Y., Cai, H.-L., Ge, J.-Z. & Xiong, R.-G. (2010). *J. Am. Chem. Soc.* **132**, 7300–7302. 10.1021/ja102573h 20459097

